# Barriers to Electronic Health Record Adoption: a Systematic Literature Review

**DOI:** 10.1007/s10916-016-0628-9

**Published:** 2016-10-06

**Authors:** Clemens Scott Kruse, Caitlin Kristof, Beau Jones, Erica Mitchell, Angelica Martinez

**Affiliations:** Texas State University, 601 University Drive, HPB rm 254, San Marcos, TX 78666 USA

**Keywords:** Barriers, Challenges, Electronic health records, Adoption: implementation

## Abstract

Federal efforts and local initiatives to increase adoption and use of electronic health records (EHRs) continue, particularly since the enactment of the Health Information Technology for Economic and Clinical Health (HITECH) Act. Roughly one in four hospitals not adopted even a basic EHR system. A review of the barriers may help in understanding the factors deterring certain healthcare organizations from implementation. We wanted to assemble an updated and comprehensive list of adoption barriers of EHR systems in the United States. Authors searched CINAHL, MEDLINE, and Google Scholar, and accepted only articles relevant to our primary objective. Reviewers independently assessed the works highlighted by our search and selected several for review. Through multiple consensus meetings, authors tapered articles to a final selection most germane to the topic (*n* = 27). Each article was thoroughly examined by multiple authors in order to achieve greater validity. Authors identified 39 barriers to EHR adoption within the literature selected for the review. These barriers appeared 125 times in the literature; the most frequently mentioned barriers were regarding cost, technical concerns, technical support, and resistance to change. Despite federal and local incentives, the initial cost of adopting an EHR is a common existing barrier. The other most commonly mentioned barriers include technical support, technical concerns, and maintenance/ongoing costs. Policy makers should consider incentives that continue to reduce implementation cost, possibly aimed more directly at organizations that are known to have lower adoption rates, such as small hospitals in rural areas.

## Introduction

### Rationale

Health information technology (HIT) and the use of electronic health records (EHRs) has increased substantially through efforts to achieve the following: reduce medical errors, provide more effective methods of communicating and sharing information among clinicians, lower national health care costs, better manage patient medical records, and improve coordination of care and health care quality [1, 2]. The Federal Government has encouraged the adoption of EHRs through incentives found within the Health Information Technology for Economic and Clinical Health (HITECH) Act, which produced Health Information Technology Regional Extension Centers (RECs). HITECH created sixty-two RECs nationwide and allocated 657 million dollars in federal funding in 2010 [3]. In addition, the Center for Medicare and Medicaid Services (CMS) has also facilitated the expansion of EHRs by providing incentives for adoption and meaningful use, and even penalties for lack of provider engagement [3]. Altogether, the United States Federal Government has invested more than twenty billion dollars to boost EHR implementation rates [2]. Various local initiatives have also emerged with the intent of further increasing adoption rates within their respective communities.

While adoption and implementation has increased significantly within the recent years, not all hospitals and healthcare organizations have chosen to adopt an EHR system. Even with the incentives offered by the Federal Government and CMS, only one out of four hospitals in 2014 had not obtained a basic EHR system [4]. Studies suggest that smaller, rural hospitals are less likely to adopt as well as practices headed by physicians over the age of 55 [3, 5, 6]. It is also suggested that EHRs are less likely to be adopted in organizations with higher populations of low-income patients and certain states [3, 6].

### Objective

The objective of this systematic literature review is to better understand the barriers that have deterred certain healthcare organizations from adopting even a basic electronic health record system in the United States. Though the most crucial factors are being identified by frequency and may not be addressed in the order of significance, this research can potentially be used by policymakers and/or future researchers.

## Methods

### Eligibility Criteria

Articles, studies, and reviews were eligible for this review if they were published in the last five years, in English, and either peer-reviewed or published in academic literature. We made the decision to include systematic reviews in order to capitalize on the wealth of information acquired previously in order to better validate our review.

### Information Sources and Search

**Fig. 1:**
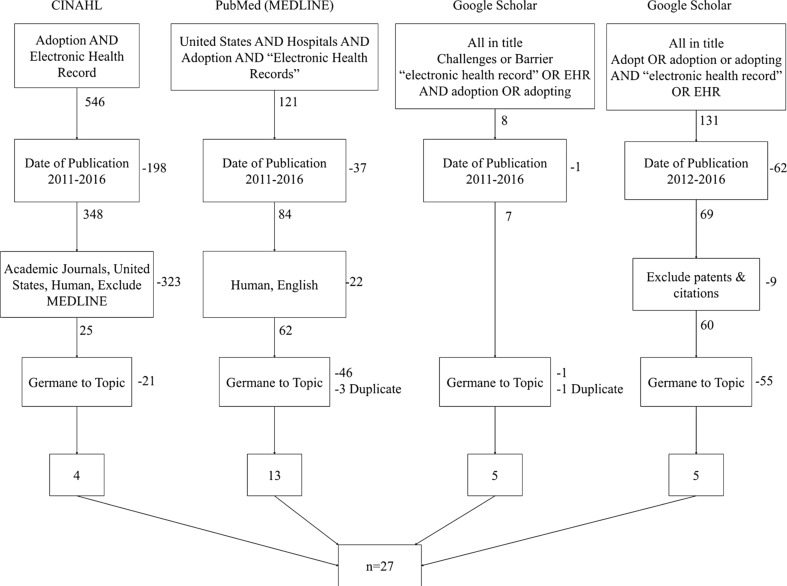
Literature search criteria with inclusion and exclusion criteria

Three databases were used to acquire articles for this review: Cumulative Index of Nursing and Allied Health Literature (CINAHL), PubMed (MEDLINE Complete) and Google Scholar. We identified articles for research based upon their relevance to the topic of barriers to adoption of EHRs. To achieve the desired field of germane articles for our research, we used key words and similar terms in conjunction with Boolean operators tailored to the search engine of the database. Fig. 1 illustrates our search methodology as well as the various search strings used to call up eligible articles.

Small terminology differences existed between research databases because the databases index differently and often use different subject headings. For instance in CINAHL, the search string was “Adoption AND ‘electronic health record’” while the search in PubMed needed to be a bit more complex. We included both studies and reviews if they met our eligibility criteria.

### Study Selection, Data Collection Process, and Data Items

Figure 1 illustrates our search process to narrow the initial result of 806 results down to the final sample of 27. As is shown, the date range for the initial search was from 1/1/2011 to 8/3/2016, with the initial date being significant as it represents the beginning of the incentives for Meaningful Use being put into effect. For the search in Google Scholar, we ended up shortening the date range to 1/1/2012 to 8/3/2016. The limited filtering capabilities in Google Scholar results in a very large number of results, many of which are false positives. We had to take some measure to reduce the number of results to something feasible to analyze.

### Risk of Bias and Additional Analysis

As illustrated in Fig. 1, after the filters were enabled the number of results was reduced to 154 possible articles. The group of reviewers divided these among them in a way that each abstract was reviewed by at least two people. A shared spreadsheet was posted to a shared collaboration site, and each reviewer independently made an assessment (1 = include, 0 = exclude) to determine if the article was germane to our review objective.

Through a set of consensus meetings, the group of reviewers met to discuss their assessments. By the end of the consensus meeting a total of 27 remained. These were then divided again in the same manner as the abstracts; each article was analyzed by at least two reviewers. The reviewers agreed to analyze the articles for possible bias, basic findings germane to our objective, and barriers that tie multiple studies together. Observations were recorded on the shared spreadsheet again and another consensus meeting was held to discuss the findings.

### Synthesis of Results and Additional Analysis

The literature matrix created by our shared spreadsheet catalogued the relevant articles by date of publication and contained fields for the following: Data source, publication date, authors, title, relevance and each rater’s decision of whether or not the article was germane to our research, general observations and specific barriers. From the data collected from the articles, we created an affinity diagram to illustrate the frequency of barriers.

## Results

### Study Selection and Characteristics

Of the 806 initial search results acquired from three databases, we isolated 27 unique publications which contained data specifically relevant to our topic. These are captured in Table 1.

**Table 1:** Analysis of articles

Authors	Barriers
Hamid, F. & Cline, T. [7]	Lack of usefulness
	Physician autonomy
	Physician attitude
	Lack of technology support
Wang, T. & Biedermann, S. [8]	Lack of capital resources to invest in EHR
	Lack of technical infrastructure
	Insufficient time
	Inability to easily input historic medical record data
	Lack of technical support staff
	Difficulty meeting eligibility criteria
Furukawa MF, et al. [9]	Technical concerns Privacy concerns
Adler-Milstein J, et al. [10]	Financial challenges (upfront and ongoing costs)
	Physician cooperation Complexity of meeting meaningful use challenges
Abramson EL, et al. [11]	Amount of capital needed
	Lack of clear state and federal/policies standards
	Concerns about ongoing maintenance costs
	Lack of adequate IT staff
	Resources for training in EHR documentation
	Resources for training in basic computer literacy
	Uncertainty about ROI
	Concerns about illegal record tampering or “hacking”
Jamoom, E. & Hing, E. [12]	Cost of purchasing a system Productivity loss Annual maintenance cost Adequacy of training Adequacy of technical support Reliability of the system Effort needed to select a system Resistance of practice to change work habits
	Ability to secure financing Reaching consensus within the practice Access to high speed internet
Menon S, et al. [13]	Safety concerns 53 % of respondents claimed an EHR safety-related event in the past five years, 10 % said they experienced 20 or more in the last 5 years Errors concerned workflow processes, familiarity & training and degree of integration
Gabriel MH, et al. [14]	Cost identified several times for critical-access and rural hospitals Workflow and staffing challenges associated with maintaining an EHR.
DesRoches CM, et al. [15]	Cost Penalties (potential future barrier)
Harle CA, et al. [16]	Lack of financial incentive mentioned as Meaningful Use was meant to overcome that particular hurdle.
Vest JR, Yoon J & Bossak BH [17]	Lack of support Costs associated with switching to EHRs Lack of incentives Difficult implementation timelines
Abramson EL, et al. [18]	Initial cost of HIT investment Lack of technical IT staff Lack of fiscal incentives Work flow challenges Lack of interoperability of EHR Cost of purchasing and maintaining an EHR system
Sockolow PS, et al. [19]	Implementation cost Training Lack of use acceptance
Reganti KR, et al. [20]	Lack of financial resources Ongoing maintenance costs Loss of productivity Increased time to document clinical info in digital format Integration
	Business impact (transitioning)
Raglan GB, et al. [21]	Consensus on selecting EHR system Finding system that meets needs Effort to select system Cost Loss of productivity System reliability and tech support Resistance to changing work habits
Simpson JL [22]	Lack of computer of typing skills
	Desire to deal with EHR
	Lack of or insufficient technical support and training from vendors complexity of system
	Limitations of system Lack of customizability to meet special practice needs (work flow issues) unreliability of system- technical glitches (ongoing maintenance costs)
	Interconnectivity and standardization challenges (incompatibility with existing systems)
	Lack of hardware (internet connectivity)
Kruse CS, et al. [23]	Cost
	Time consuming
	User perception/perceived lack of usefulness
	Transition of data
	Facility location
	Implementation issues
	User/patient resistance
	Lack of technical assistance/experience
	Interoperability/no standard protocols
Kruse CS, et al. [24]	Cost
	Time consuming
	User perception/perceived lack of usefulness
	Transition of data
	Facility location
	Implementation issues
	User/patient resistance
	Lack of tech assistance/experience
	Interoperability/no standard protocols for data exchange
	Medical error
	Training
	Maintenance
	Upgrades
	Lack of agility to make changes
	Staff shortages/overworked
	Privacy and/or security
	Missing data
	External factors
	Competitiveness
	Provider or patient age, race &income disparities, lack of infrastructure and/or space for systems
	Organizational cultural change
	Lack of incentives
	IMGs less likely to adapt
Ben-Zion R, et al. [25]	Implementation costs
	User resistance
	System usability
	Cost benefit symmetry
	Privacy
	Lack of protocol
Beasley S & Girard J [26]	Federal regulations
	Costs
	Time needed
	Eligibility criteria
Meigs S & Solomon M [27]	Interoperability
	System costs
	Workflow disruption
	Reduced productivity
	Difficult usability

From these articles we identified 68 barriers to adoption, but they varied widely in how they were originally presented.

Several of the barriers seemed to be close enough to one another to combine, so we compiled these in another table (Table 2).

**Table 2:** Frequency of barriers

Barriers	Occurrences by article reference number	Total occurrences
Initial Cost	8,10–12,14–27	18
Technical Support	7,8,11,12,14,17,18,22–24	10
Technical Concerns	9,12,18,20–25	9
Resistance to Changing Work Habits	12,13,20–25	8
Maintenance/Ongoing Costs	10,11,12,17,18,20,22,24	8
Training	11–14,19,22,24	7
Privacy Concerns	9,11,13,24,25	5
Insufficient Time	8,17,20,23,24	5
Workflow Challenges	13,14,18,21,27	5
Financial Incentives	12,16,17,18	4
Productivity loss	12,20,21,27	4
Perceived Usefulness	7,23,24	3
Inability to easily input historic medical record data	8,13,20	3
Eligibility Criteria	8,10,26	3
Technical Infrastructure	8,24	2
Physician Attitude	7,10	2
Effort Needed to Select System	12,21	2
Degree of Integration	20,22	2
Facility location	23,24	2
ROI Uncertainty	11,25	2
Clarity of Federal and State Policies	11,26	2
Complexity of system	22,27	2
Physician Autonomy	7	1
Consensus within the practice	12	1
Penalties	15	1
User acceptance	19	1
Limitations of system	22	1
Medical errors	24	1
IMGs less likely to adopt	24	1
Staff shortages	24	1
Upgrades	24	1
Agility to make changes	24	1
External factors	24	1
Missing data	24	1
Competitiveness	24	1
Provider or patient age	24	1
Race and income disparities	24	1
Need organizational cultural change	24	1
Interoperability	27	1
		120

For example, *cost*, *lack of capital resources to invest in EHR*, *financial challenges*, *cost of purchasing system*, *initial cost of HIT* and *lack of financial resources* were grouped into *initial cost*. *Complexity of meeting meaningful use criteria* and *difficulty meeting eligibility criteria* were grouped under *eligibility criteria*. *Physician cooperation* and *physician attitude* were merged under *physician attitude*. *Lack of adequate IT staff*, *lack of technical support*, *lack of technology support,* and *adequacy of tech support* were merged into *technical support*. *Concerns about illegal record tampering & hacking* and *safety concerns* were merged with *privacy concerns*. *Access to high-speed internet* and *lack of interoperability* were merged with *technical concerns*. *Errors concerned with workflow* was merged with *workflow challenges*. *Difficult implementation timelines* and *time consuming* were merged with *insufficient time*. *Lack of usefulness* and *lack of use* were merged into *perceived usefulness*. *Business impact*, *resistance to changing work habits*, *lack of desire to deal with EHR*, and *implementation issues* merged with *resistance to change*. *Integration* was combined with *degree of integration*. *Consensus on selecting EHR* combined with *reaching a consensus within the practice*. *Transition of data* incorporated into *inability to input easily historic medical record data*. *Complexity of system* and *limitations of system* were grouped together. After collapsing/combining barriers, we had 39 barriers which occurred in the literature 125 times.

### Results of Individual Studies and Additional Analysis

The *initial cost* of implementing a system is consistently considered a top, major barrier to the implementation of electronic health record systems appeared 14. 4 % of all occurrences (18/125) [8, 10–12, 14–27]. *Technical support* appeared 8.0 % of all occurrences (10/125) [7, 8, 11, 12, 14, 17, 18, 22–24]. *Technical concerns* appeared 7.2 % of all occurrences (9/125) [9, 12, 18, 20–25]. *Maintenance/ongoing costs* [10–12, 17, 18, 20, 22, 24] and *resistance to changing work habits* [12, 13, 20–25] occurred 6.4 % of all occurrences (8/125). *Training* appeared 5.6 % of all occurrences (7/125) [11–14, 19, 22, 24]. Next were *insufficient time* [8, 17, 20, 23, 24], *privacy concerns* [9, 11, 13, 24, 25], and *workflow challenges* [13, 14, 18, 21, 27] which each appeared 4.0 % of all occurrences (5/125). *Financial incentives* [13, 14, 18, 21] and *productivity loss* [12, 20, 21, 27] appeared 3.2 % of all occurrences (4/125). All the other barriers appeared three times or less among the sample, and we judged these to be of minor significance and not worth greater scrutiny.

## Discussion

### Summary of Evidence

From our sample of 27 articles, we identified 68 barriers (that we collapsed into 39) that appeared in the literature a total of 125 times. Our analysis of these articles was that initial cost appeared the most often, followed by technical support, technical concerns, resistance to changing work habits, maintenance/ongoing costs, training, privacy concerns, insufficient time, workflow challenges, financial incentives, perceived usefulness, productivity loss, inability to easily input historical medical record data, eligibility criteria, technical infrastructure, physician attitude, effort needed to select system, degree of integration, facility location, ROI uncertainty, clarity of federal and state policies, physician Autonomy, consensus within the practice, penalties, user acceptance, complexity of system, limitations of system, medical errors, IMGs less likely to adopt, staff shortages, Upgrades, agility to make changes, external factors, missing data, competitiveness, provider or patient age, race and income disparities, need organizational cultural change and interoperability.

Results from this systematic literature review are congruent with results from previous reviews. The most often cited barrier is cost, technical concerns, implementation, and user perceptions [23, 24, 26]. The HITECH act began with the carrot of incentives to adopt HIT, but now it only offers the stick of reduced reimbursements for those who do not adopt HIT. As a result, few solutions to the common barriers currently exist, making policy and incentives play a crucial role in increasing the adoption rates of EHRs in the United States.

Of interesting note is that of *interoperability.* This critical barrier only occurred in the literature once for the U.S., and only recently [27]. Several other articles were identified worldwide on the subject [28–30]. Providers have voiced their discontent with the lack of interoperability established by government agencies, which enables the static nature of EHR development at the vendor [27]. Providers are also frustrated by the lack of progress in interoperability because it greatly limits the progression of specialties such as pain management [28]. Lack of interoperability stymies efforts of information governance and information sharing between organizations [29]. Lastly, providers and programmers alike agree that greater interoperability is needed today to create standardization for data that can or will be staged for a data warehouse [30].

Policy makers should consider the results of this review when shaping additional policies and incentives for healthcare organizations in the future. This review contributes to previous findings that suggest the most common barriers revolve around cost issues, technical support, technical concerns, and resistance to change. Considering both initial and ongoing costs are ongoing barriers that may disproportionately affect certain organizations that are lagging such as practices in small and rural settings, incentives should be more directly aimed towards correcting these disparities. Policy makers should also consider ways to increase the appeal of electronic health record systems to older physicians, organizations that deal with large populations of low-income patients, and certain states that have lower adoption rates.

### Limitations

There are several limitations inherent to our review. Selection bias is always a concern when there is subjectivity involved in the screening and selection process. We attempted to control for this bias through our many consensus meetings. During these meetings we would share observations and ensure that we were all looking for the same things.

Another common bias is publication bias. Because our search was largely limited to research databases, we would only capture articles, studies, and reviews that were published, which is often based on significance of findings. We attempted to control for this bias by searching in Google Scholar. This control resulted in a great effort to screen through what we knew would be false positives, but in the end it did yield articles that were not discovered in the established research databases.

## Conclusions

With regard to future research, the healthcare industry is quickly becoming saturated with at least basic EHRs, and the segment of the industry that has not adopted will likely continue to narrow while the adoption rate sharply decreases. As a result, further research into the barriers and facilitators of adoption will benefit fewer people as time passes, and thus decrease in overall value. The most beneficial use of the research resources in this area would likely be in better cataloguing and dissemination of the more successful EHRs, their implementation and manner of upgrading with minimal disruption of standard operations. Additionally, further research in the area of EHRs would likely profit from identifying key factors of achieving the current stages of meaningful use in an effective manner, or according to the targets of the oncoming Merit-Based Incentive Payment System (MIPS) under the Medicare Access and CHIPS Reauthorization Act (MACRA). This in particular would be most beneficial as we believe that many healthcare institutions, as shown by the research, fail to achieve meaningful use as they have adopted an excessively basic EHR in order to meet the minimum standards for regulation under HITECH. This low-functioning EHR can actually negatively impact the time, effort and cost as opposed to streamlining processes, reducing error and reducing cost, which are the hallmarks of a high-functioning EHRs. Indeed, the benefits of a high-functioning EHR and the methods of achieving this could use additional illumination.

### Authors Contributions

Clemens Scott Kruse, Beau Jones, Erica Mitchell, Caitlin Kristof, and Angelica Martinez meet the ICMJE definition of authorship:

Clemens Scott Kruse, Beau Jones, Erica Mitchell, Caitlin Kristof, and Angelica Martinez made substantial contributions to the conception or design of the work; or the acquisition, analysis, or interpretation of data for the work; AND.

Clemens Scott Kruse, Beau Jones, Erica Mitchell, Caitlin Kristof, and Angelica Martinez helped in drafting the work or revising it critically for important intellectual content; AND.

Clemens Scott Kruse, Beau Jones, Erica Mitchell, Caitlin Kristof, and Angelica Martinez have given final approval of the version to be published; AND.

Clemens Scott Kruse, Beau Jones, Erica Mitchell, Caitlin Kristof, and Angelica Martinez are in agreement to be accountable for all aspects of the work in ensuring that questions related to the accuracy or integrity of any part of the work are appropriately investigated and resolved.
